# Pre-Exercise Factors Associated with the Magnitude of Exercise-Induced Hypoalgesia in Individuals with Knee Osteoarthritis: A Cross-Sectional, Observational Study

**DOI:** 10.3390/jcm14228086

**Published:** 2025-11-14

**Authors:** David Toomey, Gwyn Lewis, Jo Nijs, Usman Rashid, Natalie Tuck, David Rice

**Affiliations:** 1 Department of Physiotherapy, School of Allied Health, Auckland University of Technology, Auckland 0627, New Zealand; gwyn.lewis@aut.ac.nz (G.L.); usman@smme.edu.pk (U.R.); natalietuck@gmail.com (N.T.); david.rice@aut.ac.nz (D.R.); 2 Pain in Motion Research Group (PAIN), Department of Physiotherapy, Human Physiology and Anatomy, Faculty of Physical Education & Physiotherapy, Vrije Universiteit Brussel, 1050 Ixelles, Belgium; jo.nijs@vub.be; 3 Unit of Physiotherapy, Department of Health and Rehabilitation, Institute of Neuroscience and Physiology, Sahlgrenska Academy, University of Gothenburg, SE-405 30 Gothenburg, Sweden; 4 PijnPraxis.be Private Practice for Pain Physiotherapy, 3970 Leopoldsburg, Belgium; 5 Waitematā Pain Services, Department of Anaesthesiology and Perioperative Medicine, Te Whatu Ora Waitematā, Auckland 0622, New Zealand

**Keywords:** osteoarthritis, musculoskeletal pain, rehabilitation medicine, physiotherapy, psychology, non-pharmacological, exercise induced hypoalgesia

## Abstract

**Background**: The magnitude of exercise-induced hypoalgesia (EIH) varies across individuals with knee osteoarthritis (OA). Impaired EIH may limit the pain-relieving effects of exercise and reduce exercise adherence. This study aimed to identify key factors associated with EIH in knee OA. **Methods**: This cross-sectional study included 119 participants (mean age 68 ± 10) with knee OA. Pre-exercise assessments, including validated questionnaires and quantitative sensory testing were undertaken. EIH was evaluated by measuring pressure pain thresholds (PPT) at the knee and forearm before and after quadriceps isometric resistance exercise. Linear regression and mixed models were used to identify factors associated with the magnitude of EIH and sources of variance in EIH. **Results**: EIH was greater at the knee compared to the forearm (*p* < 0.01), with considerable inter-individual variability. Older age, less anxiety, and expecting less exercise-induced pain were associated with increased EIH (all *p* < 0.05). However, all measured variables explained <20% of the variance in EIH, with unobserved between-participant factors estimated to account for ≥45% additional variance. **Conclusions**: Age, pre-exercise anxiety, and pain expectations are associated with the magnitude of EIH after resistance exercise in people with knee OA. However, the contribution of these factors was modest, with much of the inter-individual variance in EIH remaining unexplained.

## 1. Introduction

Knee osteoarthritis (OA) is a prevalent musculoskeletal pain condition with substantial personal and socioeconomic burden [1]. OA is multifactorial, reflecting interacting biomechanical, occupational, and sport exposures, prior injury, adiposity, and sex-related differences, with cartilage biomechanics central to symptom development and early detection challenges [2]. Exercise is recommended as the first-line treatment for knee OA by international evidence-based guidelines [3,4]. Group-level data show that structured exercise of ≥12 sessions improves pain and physical function in knee OA [5,6], although individual responses frequently vary [7,8,9]. Particularly in the early stages of an exercise programme, some individuals experience exercise-induced flares in pain, which can reduce adherence and compromise long-term gains [8,10].

In healthy, pain-free populations, a single session of exercise typically results in exercise-induced hypoalgesia (EIH)—a reduction in pain sensitivity lasting for up to 30 min [11,12]. EIH is typically measured as the pre- to post-exercise change in pressure pain thresholds (PPT), and can occur both locally, close to the exercising muscle group(s), and remotely, at distant body sites, with larger effects frequently observed at the local site [12]. 

In people with chronic pain, including knee OA, the EIH response is less consistent [12]. Studies in people with knee OA variably demonstrate a normal EIH response [13,14,15,16], unchanged pain sensitivity [17] or an increase in pain sensitivity (i.e., exercise-induced hyperalgesia) [16,18,19]. Notably, in clinical populations, exercise-induced hyperalgesia has been associated with flares in pain [20,21,22]. In addition, variability in the long-term pain-relieving effects of exercise may be related to impaired EIH [16], with recent research in people with knee OA finding that reduced EIH, measured at baseline, predicts less improvement in pain and function after 12 exercise sessions [23].

Factors underlying the variability in EIH remain poorly understood. Most research has been in healthy, pain-free populations, with inconsistent findings [24,25,26]. Only three studies have specifically investigated factors associated with EIH in people with knee OA [14,16,22]. Kosek et al. [14] demonstrated that EIH did not differ by sex. Wideman et al. [22] found that increased knee pain after a standardised bout of walking was associated with greater pain catastrophising, greater temporal summation of pain, and raised scores on clinical questionnaires, all measured before exercise. Fingleton et al. [16] reported that individuals with intact conditioned pain modulation (CPM), a measure of endogenous pain inhibition, demonstrated a normal EIH response. In contrast, those with deficient CPM experienced exercise-induced hyperalgesia after both aerobic and resistance exercise. 

The individual variability in both EIH responses and the pain-relieving effects of exercise in people with knee OA highlights the need for further study. Understanding which factors are associated with EIH may provide important treatment targets for intervention studies that aim to improve adherence and maximise the long-term benefits of exercise in people with knee OA.

While several studies have investigated potential predictors of EIH in knee OA [14,16,22], most have been limited by small sample sizes, focused on either psychological or physiological factors alone, or have not quantified how much inter-individual variability these factors explain. Furthermore, no prior study has applied variance decomposition to estimate the relative contribution of observed and unobserved factors to EIH magnitude.

Evidence remains limited on which pre-exercise factors explain the individual variability of EIH in knee OA. Given that exercise is first-line care and EIH may influence adherence, dosing, and outcomes, we conducted an exploratory secondary analysis in a single cohort to evaluate psychological, neurophysiological, and expectancy-related factors. Using variance decomposition, we estimated the proportion of EIH variance attributable to measured versus unmeasured factors. Our primary aim was to identify modifiable pre-exercise factors associated with EIH magnitude after a sustained submaximal isometric quadriceps contraction.

## 2. Materials and Methods

This cross-sectional study was an exploratory secondary analysis of baseline (pre-intervention) data collected from a clinical trial comparing two different types of serotonin–noradrenaline reuptake inhibitors for the treatment of knee OA pain (Australia New Zealand Clinical Trials Registry: ACTRN12619001082190). The original protocol and secondary analysis were approved by the New Zealand Central Health and Disability Ethics Committee (19/CEN/27). The Strengthening the Reporting of Observational studies in Epidemiology (STROBE) was used as a guideline for reporting [27]. All assessments used in this analysis were conducted prior to the administration of study medication. Therefore, no participants had received pharmacological treatment related to the parent trial at the time of data collection.

### 2.1. Participants

Participants were recruited through online and print advertisements and from orthopaedic first surgical appointments at local hospitals in the Auckland region. Inclusion criteria for the primary study were men and women ≥40 years of age who had radiographic knee OA and met the American College of Rheumatology clinical criteria for the diagnosis of knee OA. Individuals were excluded if they were currently using antidepressant medication or any other medication with a serotonergic effect that could not be discontinued; had used a monoamine oxidase inhibitor in the last 14 days; had narrow-angle glaucoma; had a body mass index (BMI) ≥ 40; had a diagnosis of any other type of arthritis; had joint injection(s) or surgery on the index knee within the last 3 months, or were scheduled for surgery in the next 3 months; medical contraindication to the use of acetaminophen and non-steroidal anti-inflammatory drugs (NSAIDs); current use of warfarin; women who were pregnant, breast feeding or planning to become pregnant; a previous diagnosis of major psychiatric disorder; history of alcoholism; alanine aminotransferase or aspartate aminotransferase > 100 IU/L or total bilirubin > 27.4 umol/L; a glomerular filtration rate of <30 mL/min; a history of myocardial infarction in the last 12 months, QTc interval > 500 ms: unstable coronary artery disease or recent unexplained cardiac symptoms; uncontrolled hypertension (>140 mmHg systolic or ≥90 mmHg diastolic BP); a history of seizures; a history of multiple falls in the last 12 months; or an inability to perform psychophysical sensory testing, e.g., due to documented sensation changes. 

### 2.2. Sample Size

Given the exploratory nature of this study, a formal sample size calculation was not conducted. However, the included 119 participants from the main study provide sufficient power to explore the unique contribution of each of the 13 independent variables [28].

### 2.3. Procedures

**Figure 1:**
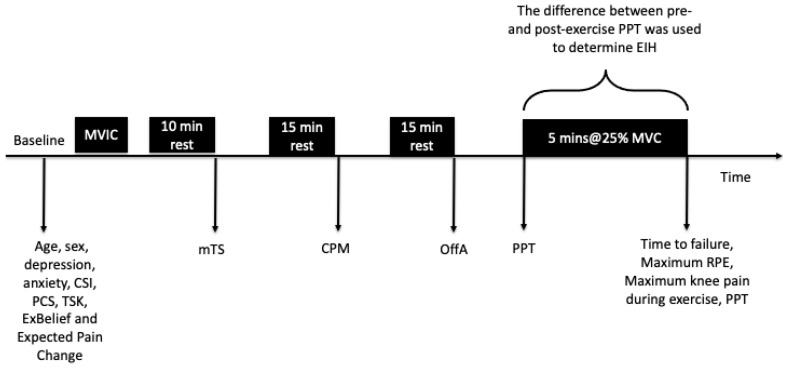
Experimental protocol, including the timing of data collected. Abbreviations: MVIC, maximum voluntary isometric contraction; mTS, mechanical temporal summation; CSI, Central Sensitisation Inventory; CPM, conditioned pain modulation; OffA, offset analgesia; PCS, the Pain Catastrophising Scale; PPT, pressure pain threshold; TSK, The Tampa Scale of Kinesiophobia; ExBelief, Beliefs About Exercise and Pain Single Item Questionnaire; EIH, exercise-induced hypoalgesia; RPE, rating of perceived exertion.

Participants abstained from caffeine and analgesic medications for 12 h prior to testing. Before assessing EIH, participants completed a series of questionnaires and underwent quantitative sensory testing (see Figure 1), as described below. The Lower Limb Task Questionnaire (LLTQ) [29], and the Brief Pain Inventory (BPI) [30] were also collected, for descriptive purposes. 

### 2.4. Dependent Variables

The dependent variables for this study were the absolute EIH (EIH_abs_) and relative EIH (EIH_rel_), determined by the change in PPT from immediately before (PPT_pre_) to immediately after (PPT_post_) quadriceps isometric resistance exercise [11,31]. PPT_pre_ was assessed at two sites, in a random order: locally at the ipsilateral medial joint line (3 cm medial to the midpoint on the medial edge of the patella) and remotely at the volar surface of the contralateral forearm (5 cm distal to the lateral epicondyle) [16]. The same test-site order was used for PPT_post_. PPTs were assessed using a handheld pressure algometer (SbMedic, Solna Sweden) with a 1 cm rounded tip and a ramping rate of 30 kpa/s. Participants were instructed to press a button the moment they first experienced any pain and the pressure in kPa (PPT) was recorded. Three PPT measurements were recorded and the average taken. EIH_abs_ was defined as the numerical difference between PPT_post_ and PPT_pre_, such that positive values reflect an increase in PPT (hypoalgesia) following exercise, while negative values reflect decreased PPT (hyperalgesia). EIH_rel_ was a ratio of PPT_post_ to PPT_pre_, such that values greater than 1.0 reflect an increase in PPT (hypoalgesia) following exercise, while values less than 1.0 reflect decreased PPT (hyperalgesia) [14]. 

#### Isometric Exercise Protocol

To allow the individualisation of quadriceps isometric exercise intensity, participants first completed an assessment of their maximum voluntary isometric contraction (MVIC) on the most symptomatic (index) knee, defined as the knee with the greatest self-reported pain during daily activities. MVIC testing was performed using a Biodex Multi-Joint System 3 Pro dynamometer (Biodex Medical Systems, Shirley, NY, USA), with the hip and knee positioned at 85° and 90° of flexion, respectively. A standardised warm-up included four 5 s isometric contractions at 25%, 50%, 50%, and 75% of perceived maximum effort, followed by three 5 s MVICs with a 30 s rest between each contraction. Consistent verbal encouragement was provided, and MVIC was defined as the peak knee extension torque (Nm) achieved during any of the 3 MVICs.

To evaluate EIH, a target isometric quadriceps torque of 25% of MVIC was selected, due to the established effectiveness of sustained submaximal isometric exercise in producing EIH [11], including in previous knee OA studies [14]. Participants maintained this torque until failure (defined as an inability to sustain the target torque for ≥5 s) or a maximum of 5 min, in which case time to failure was recorded as 300 s [14]. 

### 2.5. Independent Variables

#### 2.5.1. Clinical Variables

##### Age and Sex

Participants’ age and sex were captured as part of baseline documentation.

##### Order of PPT Testing

The order of PPT tests (knee or forearm first) was recorded.

##### Time to Failure (Seconds)

During the isometric exercise protocol, the time taken to reach true contraction failure was recorded in seconds.

##### Maximum Rating of Perceived Exertion (RPE)

During the isometric exercise protocol, a rating of perceived exertion (RPE) on Borg’s 6–20 scale [32], with 6 defined as “no exertion at all” and 20 as “maximal exertion”, was obtained every 30 s. The maximum RPE obtained during isometric exercise was recorded. 

##### Maximum Knee Pain During Exercise

Immediately after completing the isometric exercise protocol, participants were asked to rate the maximum knee pain they experienced at any time during the task, on a scale from 0 to 100, where 0 = no pain at all and 100 = the worst pain imaginable [33]. 

#### 2.5.2. Neurophysiological Variables

##### Conditioned Pain Modulation

Conditioned pain modulation (CPM) is a measure of endogenous pain modulation that reflects the balance between descending inhibitory and facilitatory pain pathways [34]. CPM was assessed following the protocol described by Yarnitsky et al. [35], using a Pathway ATS thermal stimulator (30 mm × 30 mm thermode; Medoc Ltd., Ramat Yishai, Israel) and a temperature-controlled water bath (Contherm Scientific, Lower Hutt, New Zealand). The test stimulus (Pain60) was determined by applying heat to the volar surface of the non-dominant forearm, beginning at 32 °C and increasing at a rate of 1 °C/s until the participant reported a pain intensity of 60/100 on a numerical rating scale. A safety cut-off of 50 °C was applied. The Pain60 temperature was then applied for 30 s, during which continuous pain ratings were recorded using an electronic visual analogue scale (eVAS). Following a 15 min rest, the conditioning stimulus was delivered by immersing the contralateral hand in a 46.5 °C circulating hot water bath for 60 s. The test stimulus was reapplied after 30 s of immersion, and CPM was calculated as the difference in mean pain ratings during the test stimulus alone and during concurrent application with the conditioning stimulus. The peak pain intensity of the conditioning stimulus was also recorded. 

##### Offset Analgesia

Offset analgesia (OffA) is another measure of endogenous pain modulation, characterised by a disproportionate decrease in pain perception following a slight decrease in noxious stimulation intensity [36]. Two test and two control trials were performed in a randomised order using the Pathway ATS thermal stimulator (Medoc, Israel). For the test trials, OffA was elicited using a three-stimulus heat pain paradigm consisting of T1 = Pain50 (temperature that induces pain ratings of 50 on a 0–100 scale; 5 s), T2 = Pain50 + 1 °C (5 s), and T3 = Pain50 (20 s). An eVAS was used to provide a continuous measure of pain intensity during these 30 s. The control trials used the same T1 stimulus (Pain50) at a constant temperature for 30 s while also assessing pain intensity continuously on the eVAS [36]. The difference in the average peak-to-peak decrease in pain rating from T2 to T3 between the two test and two control trials was taken as a measure of offset analgesia [36]. 

##### Mechanical Temporal Summation

Mechanical temporal summation (mTS) is a dynamic psychophysical test of pain facilitation [37]. mTS was examined using a 180 g (# 6.45) Von Frey Monofilament (North Coast Medical, Gilroy, CA, USA) over the volar forearm [35]. A single stimulus was applied, followed by 10 repetitive stimuli, with an inter-stimulus interval (ISI) of 1 s applied within an area 1 cm in diameter. The difference in pain intensity (0–100 NPRS) between the last (10th) stimulus of the sequence and the single stimulus was taken as the measure of mTS [35].

##### Central Sensitisation Inventory (CSI)

The CSI is a reliable self-report questionnaire that has been validated and demonstrates good psychometric properties [38,39]. It consists of 2 parts, of which part A is a self-report questionnaire that includes 25 items about the frequency of central sensitisation-related symptoms, scored on a 5-point Likert scale ranging from 0 to 4 [40]. Greater total scores are taken to indicate a greater burden of central sensitisation-related symptoms. Previous research provided CSI severity levels as a guideline for interpreting CSI scores: subclinical = 0 to 29; mild = 30 to 39; moderate 40 to 49; severe 50 to 59; and extreme = 60 to 100 [41]. 

#### 2.5.3. Psychological Variables 

##### Hospital Anxiety and Depression Scale (HADS)

The HADS is a 14-item questionnaire used to assess anxiety and depression symptoms. The anxiety and depression subscales are scored from 0 to 21, with larger scores indicating more symptoms [42]. This widely used instrument shows adequate validity and reliability in various populations, including people with OA [42,43,44,45].

##### The Pain Catastrophising Scale (PCS)

The 13-item PCS was used to assess catastrophic thinking related to pain, with total scores ranging from 0 to 52 [46]. The PCS has adequate internal consistency, validity, and test–retest reliability in both pain-free individuals and people with chronic pain [47,48].

##### The Tampa Scale of Kinesiophobia (TSK) 

The TSK is a 17-item questionnaire used to assess fear of movement. The degree of agreement with each item is rated on a five-point scale, with total scores ranging from 17 to 68. Larger scores reflect greater fear of movement or re-injury. This tool has been previously utilised in people with knee OA and shown to be valid and reliable [49].

##### Beliefs About Exercise and Pain (ExBelief) 

Exercise beliefs were assessed using the single questionnaire item from Jones et al. [50] specifically related to EIH, “Pain can be reduced from just a single session of exercise”, scored on a 7-point Likert scale from 0 = “strongly disagree” to 6 = “strongly agree”. Scores on this scale have previously been associated with EIH magnitude in a pain-free population [41].

##### Expected Pain Change

Immediately after MVIC testing, participants were familiarised with the isometric exercise task to obtain pain expectancy scores. Participants first described their current resting knee pain (0–100 NPRS) and were told that later in the testing session, they would be asked to hold a 25% MVIC for as long as possible, up to a maximum of 5 min. They then performed a brief 10 s isometric quadriceps contraction at 25% MVIC and were asked what they expected their knee pain to be on the same 0–100 NPRS scale after completing the same task to failure. Expected pain change was defined as expected post-exercise knee pain intensity minus resting knee pain intensity.

### 2.6. Statistical Analysis

Due to their non-normal distribution, Wilcoxon signed-rank tests were used to determine whether PPT_post_ was different from PPT_pre_ at the local (knee) and remote (forearm) sites. Thereafter, a primary statistical analysis was conducted to evaluate which independent variables were associated with the magnitude of EIH. This question was answered in terms of both EIH_abs_ and EIH_rel_ at both the knee and forearm. Thus, four linear regression models were constructed, two for each test site. The models regressed EIH_abs_ and EIH_rel_ on age, sex, CPM, OffA, TS, max RPE, depression, anxiety, PCS, TSK, ExBelief, Expected Pain Change, and the order of PPT tests (knee first, forearm first). All variables were entered as continuous, except sex and order of PPT tests, which were entered as dichotomous. The model coefficient, standard error, t-value, degrees of freedom, and *p*-value were used to evaluate the relationships between EIH and the independent variables. 

A secondary statistical analysis was conducted to examine the extent to which EIH magnitude was explained by the observed and unobserved variables. To answer the second question for each test site (knee, forearm), the absolute values of EIH*_i_* (3 repetitions per location) were fitted with linear mixed models. EIH*_i_* for the *i*-th repetition was obtained by normalising the *i*-th post-exercise PPT value by the average of the three pre-exercise PPT values, or PPT_pre_. Although not an established method for analysing EIH data, this resulted in three post-exercise EIH data points per individual instead of one. Having multiple data points allowed us to utilise variance decomposition to explore the potential sources of variability in the dependent variable (EIH_abs_). In these linear mixed models, EIH*_i_* was once again regressed on the observed variables. To account for differences across PPT repetitions, the models included repetition number as a continuous variable. Moreover, the models estimated between-participant variance. Independent variables found to have a statistically significant relationship with EIH*_i_* were reported. Using a hierarchical two-step approach along with decomposition of variance components from the linear mixed models, the proportion of variance explained by the observed variables, between-participant variance, and residual (remaining/error) variance were calculated. The between-participant variance can be attributed to unobserved participant-level variables. The residual variance may be attributed to instrument imprecision and inadequate data modelling, such as non-linear relationships between the investigated variables and EIH magnitude [51,52,53,54]. All models were fitted in R (version 4.3.1; R Foundation for Statistical Computing, Vienna, Austria). The assumptions of normality and homogeneity of variance for model residuals were evaluated with QQ-plots and fitted-values versus residuals plots. The presence of multicollinearity was assumed to be at a variance inflation factor (VIF) of 10, and if observed, variables were excluded based on expert judgement [55]. An alpha level of *p* < 0.05 was used for all statistical tests.

## 3. Results

### 3.1. Participant Characteristics

A total of 129 individuals provided written informed consent and began data collection. Ten participants were subsequently excluded: eight due to equipment failure that precluded the collection of all QST measures, and two upon the discovery of undisclosed neurological conditions during data collection procedures that may have affected the validity of QST measures. Consequently, the data of 119 participants (92% of the initial cohort) were included in the final analysis (Table 1).

**Figure 2:**
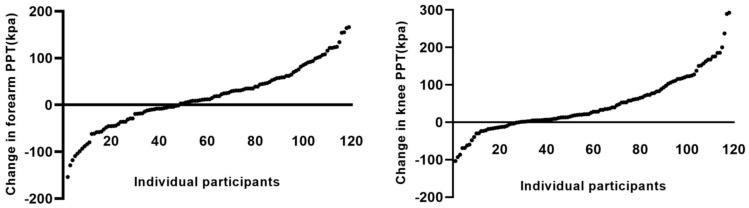
Distribution of the absolute change in knee pressure pain thresholds from pre- to post-isometric exercise of the quadriceps for individual participants at the forearm (remote site, left plot) and at the knee (local site, right plot), ordered from the most hyperalgesic (**left**) to the most hypoalgesic (**right**) response.

At the group level, a significant increase in local (knee) and remote (forearm) PPT was observed from pre- to post-exercise, indicating an overall exercise-induced hypoalgesic response (Table 2). Despite this, there was notable inter-individual variability in EIH (Figure 2), with *n* = 48 (40%) and *n* = 27 (23%) showing no change in PPT or reduced PPT (i.e., exercise-induced hyperalgesia) at the forearm and knee, respectively.

### 3.2. Factors Associated with EIH Magnitude at the Knee and Forearm

#### 3.2.1. Relationships Between Independent Variables and Absolute EIH 

Model residuals were all normally distributed, and VIF values were all <3, with no variables excluded based on multicollinearity. No significant association was observed between any of the independent variables and EIH_abs_ at the knee (all *p* > 0.06). EIH_abs_ at the forearm demonstrated a significant association with pre-exercise HADS-anxiety, such that a one-unit increase in anxiety corresponded with a decrease in EIH_abs_ of 7.1 ± 2.8 kPa (*p* = 0.01).

#### 3.2.2. Relationships Between Independent Variables and Relative EIH 

When expressed as EIH_rel_, HADS-anxiety and age had a significant association at the forearm. Each one-unit increase in pre-exercise HADS-anxiety was linked to a decrease of 0.022 ± 0.009 in the EIH ratio at the forearm (*p* = 0.02). Each additional year of age was associated with an increase of 0.006 ± 0.003 in the EIH ratio (*p* = 0.04). For EIH_rel_ at the knee, age was again positively associated with EIH ratio, where each additional year of age was associated with a 0.007 ± 0.003 increase in the EIH ratio (*p* = 0.02).

#### 3.2.3. Sources of Variance in EIH Magnitude at the Knee and Forearm

Linear mixed-effects models revealed a significant relationship between EIH_abs_ at the knee and two independent variables: age and expected pain change. Specifically, age was positively associated with EIH_abs_ at the knee (*b* = 0.007, *SE* = 0.003, *t*(119) = 2.581, *p* = 0.01), indicating that older participants had greater EIH. Conversely, the expected change in pain was negatively associated with EIH (*b* = −0.002, *SE* = 0.0009, *t*(119) = 2.010, *p* = 0.047), indicating that participants expecting greater increases in knee pain with exercise had less EIH. 

**Figure 3:**
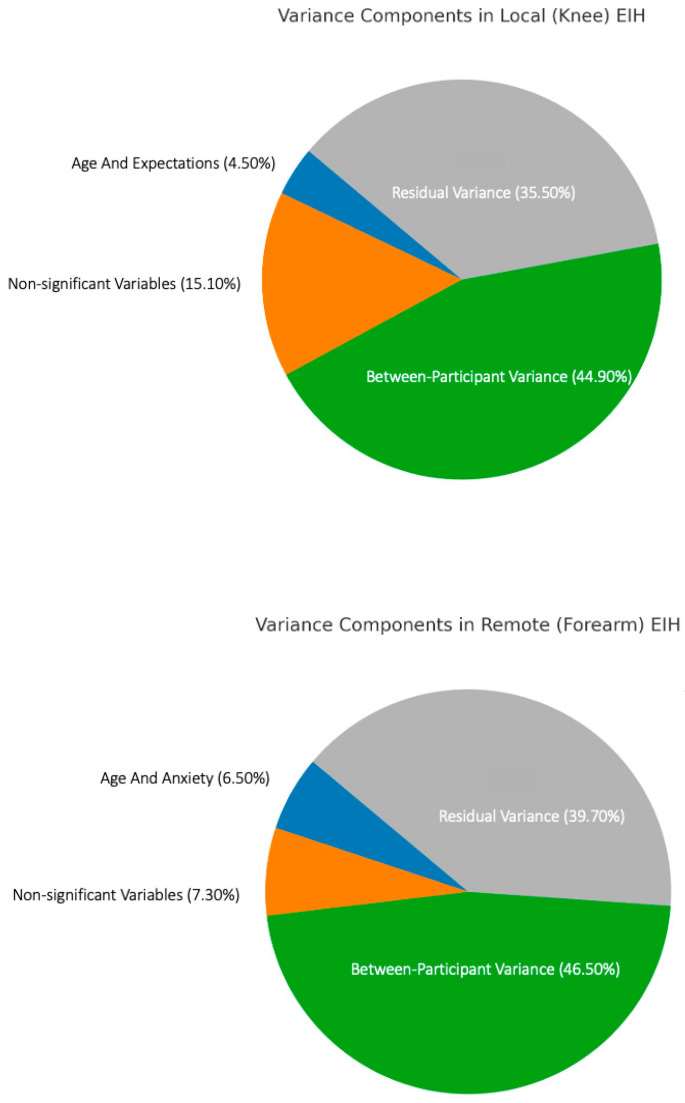
Proportions of variance in EIH magnitude at the knee and forearm.

Variance decomposition indicated that 4.5% of the variance was accounted for by age and expected pain change. Notably, 15.1% of the EIH variance was explained by non-significant observed variables and differences across repetitions, with a substantial 44.9% attributable to unobserved between-participant characteristics. Residual variance was 35.5% (Figure 3).

In terms of EIH_abs_ at the forearm, model coefficients for age (*b* = 0.005, *SE* = 0.003, *t*(119) = 2.234, *p* = 0.027) and anxiety (*b* = −0.022, *SE* = 0.009, *t*(190) = −2.537, *p* = 0.012) were significant, indicating that the magnitude of forearm EIH was greater in participants who were older and had reduced pre-exercise HADS-anxiety scores. Variance decomposition showed that age and anxiety uniquely explained 6.5% of the variance in EIH. An additional 7.3% of the variance was explained by non-significant observed variables and differences across repetitions. Again, the largest proportion of variance, 46.5%, was due to unobserved between-participants characteristics, with residual variance making up the remaining 39.7% (Figure 3).

## 4. Discussion

There is a clear gap in the literature regarding which pre-exercise factors best explain individual variability in EIH in people with knee OA. Clarifying these factors is clinically important because exercise is a first-line treatment for knee osteoarthritis, yet hypoalgesic responses vary widely and may influence adherence, dosing, and outcomes. The primary purpose of this study was therefore to identify clinical, psychological, and neurophysiological pre-exercise factors associated with EIH magnitude in people with knee OA. Furthermore, variance decomposition was utilised to estimate how much variability was attributable to measured versus unmeasured factors.

In this study of 119 people with knee OA, we observed statistically significant increases in PPTs at both the knee (local) and forearm (remote) following a sustained submaximal isometric quadriceps exercise, indicating an overall EIH response at the group level.

Unlike previous studies that examined single domains of influence on EIH [14,16,22] the present analysis integrated psychological (e.g., anxiety, catastrophising, kinesiophobia, expectations) and neurophysiological (CPM, offset analgesia, mechanical temporal summation) factors within the same cohort. Importantly, we applied variance decomposition, a novel approach in this field, to estimate the proportion of EIH variance explained by measured variables versus unobserved participant-level factors.

By quantifying the relative and absolute contributions of these domains, this study extends earlier work beyond simply identifying correlates of EIH to providing a data-driven estimate of how much EIH variability remains unexplained. This approach helps refine mechanistic hypotheses and directs attention toward additional systems (e.g., autonomic, immune, or genetic) that may further account for the observed variability.

The findings of this study align with previous studies reporting an indication of overall exercise-induced hypoalgesic response at the group level in people with knee OA [13,14,56]. However, as observed in previous studies [17,18,19,23], substantial variability in EIH responses was observed at the individual level, with 23 and 40% of participants showing no change or an increase in local and remote pressure pain sensitivity post-exercise, respectively.

This study aimed to identify pre-exercise factors associated with individual differences in EIH magnitude and quantify the sources of variance contributing to EIH. Across all models, age was positively associated with EIH magnitude, while HADS-anxiety and expected change in pain were negatively associated. Increasing age was associated with greater EIH at both the knee and forearm, particularly when expressed as EIH_rel_. Each decade of age corresponded to ~7% and ~6% increases in forearm and knee EIH, respectively. This contrasts with some prior studies showing no age-related variation in EIH [57], or reduced EIH in older adults [58]. The discrepancy may reflect differences in populations tested and/or methodology. For example, lower baseline PPTs in older adults [59] may result in proportionally larger relative changes post-exercise. Although our models adjusted for baseline PPTs, some influence may remain. Furthermore, Vaegter et al. [57] included only participants under 65, while Naugle et al. [58] compared two distinct age groups, young adults (19–30 years) and older adults (55–74 years), including a much younger cohort and age range than the current study. Differences in covariate inclusion between studies could also explain some variation in findings. For example, Ohlman et al. [26], have shown that greater physical activity levels predict greater EIH in older adults (60–77 years), implying that physically active older adults may retain better pain modulatory capacity. These findings emphasise the importance of considering additional covariates that may interact with age when investigating EIH in people with chronic pain conditions, including knee OA.

Increased pre-exercise HADS-anxiety scores were linked to a reduced EIH at the forearm. These findings contrast with previous studies generally showing no association between anxiety and EIH [24,60,61,62,63,64,65]. Anxiety may lead to a reduction in endogenous opioid function, thought to play a key role in mediating the EIH response [11,66]. For example, anxiety is associated with increased cholecystokinin (CCK) release, an opioid antagonist, and reduced opioid receptor function [11,66]. Anxiety has also been associated with higher levels of circulating inflammatory markers (e.g., IL-6, CRP), which can enhance pain sensitivity and reduce pain modulation capacity [67,68]. It should be noted that the median (IQR) anxiety score on the HADS was 5 (3–7) in our sample, indicating that most of our participants were experiencing mild symptoms of anxiety [69]. As such, the effect of more severe anxiety on EIH in people with knee OA remains uncertain.

In contrast to Wideman et al. [22], we found no association between pain catastrophising and EIH. A possible explanation is a floor effect: median PCS in our sample was 10/52 (IQR 5–17), well below the ≥30 threshold often considered clinically meaningful. Previous work shows that catastrophising moderates EIH only when baseline scores are higher, and that situational (state) catastrophising rather than dispositional PCS may be the stronger predictor of post-exercise hypoalgesia [24].

In the current study, a higher expected increase in knee pain was associated with a smaller EIH response at the index knee. Expectations are well-known modulators of both experimental pain and clinical outcomes, and pre-exercise messaging that shapes those expectations can markedly alter the magnitude of EIH. Two randomised controlled trials in healthy, pain-free participants [50,70] have shown that pre-exercise information designed to influence pain expectations can significantly alter the EIH response. In one study [70], participants who received positive information about the pain-relieving effects of exercise experienced a 22% increase in PPT at the exercising muscle after a 3 min isometric contraction, while those given negative information experienced a 4% decrease in PPT. Similarly, Jones et al. [50], reported that positive pre-exercise education enhanced EIH relative to neutral messaging. Both studies also found significant correlations between expectation ratings and EIH magnitude.

Our findings extend previous work by demonstrating, for the first time to our knowledge, a direct association between pre-exercise pain expectations and EIH magnitude in people with knee OA. Notably, the median expected change in knee pain in our sample was 45 on a 0–100 NPRS, whereas the median actual change was 0. This stark discrepancy highlights how participant expectations regarding pain relief were markedly overestimated.

This misalignment may be clinically meaningful, as overly negative expectations could contribute to a blunting of the hypoalgesic response. While the cross-sectional nature of our study precludes causal inference, experimental studies have shown that pain-related expectations can directly influence pain outcomes and hypoalgesia [50,70]. Given that exercise is a first-line treatment for knee OA, brief, targeted strategies to align or optimise expectations, alongside approaches to reduce anxiety may offer pragmatic, modifiable levers to enhance EIH and support adherence and outcomes in clinical care. 

Consistent with prior work [14], no association was observed between sex and the magnitude of EIH. Unlike Wideman et al. [22], we found no association between TS and EIH, perhaps because TS was measured at the forearm. Wideman et al. [22] found a significant relationship to EIH only when TS was measured at the painful knee, not at a remote site. We also could not replicate the association between EIH and CPM reported by Fingleton et al. [16]. While the EIH methods in our studies were very similar, the CPM paradigms differed. We used a thermal test stimulus (heat) and a painfully hot conditioning stimulus, presented in parallel [35]. In contrast, Fingleton et al. [16] assessed the change in PPT serially, after a painfully cold conditioning stimulus. There is growing evidence that different CPM paradigms may produce varied results that at least in part reflect distinct underlying mechanisms [71,72].

To our knowledge, this is the first study to use variance decomposition to estimate the sources of variability in EIH. Across models, all the variables we measured explained less than 20% of EIH variance. These results are consistent with previous findings exploring sources of variability in CPM. Graeff et al. [73] reported that for CPM, unexplained between-participant differences accounted for 24–34% of the variance, while commonly measured variables such as age, sex, and conditioning stimulus intensity collectively explained less than 12% of CPM variance. In the current study, unexplained between-participant differences accounted for ≥45% of the variance, highlighting the need to explore additional factors contributing to EIH variability in future research. This could include autonomic nervous system function [74], especially in less active individuals [75], immune responses to exercise [12], and differences in genotype [76] that may influence EIH. Furthermore, pain in knee OA arises from whole-joint pathology involving coordinated activity of chondrocytes, osteoblasts, synoviocytes, and immune cells through inflammatory and growth-factor signalling [77]. Whether variability in these cellular and molecular mechanisms contributes to the between-person differences in EIH observed here is unknown. The modest proportion of variance explained by our measured pre-exercise factors (Figure 3) suggests that other unmeasured biological variables could account for some of the observed heterogeneity. Future mechanistic studies integrating clinical phenotyping with molecular profiling are needed to elucidate potential biological substrates of EIH responsiveness in knee OA. Finally, the contribution of residual (unexplained) variance was 36–40% across models. This may be attributed to instrument imprecision and/or inadequate data modelling, such as non-linear relationships between some of the variables and EIH magnitude, that could be explored further in future research. 

This study has several strengths, including the large number of variables measured, the inclusion of people with both radiographic and clinical evidence of knee OA, the large sample size, standardisation of testing protocol, the use of variance decomposition to estimate sources of EIH variability, and the careful measures taken to reduce bias due to confounding variables such as analgesic intake. There are also some potential limitations to consider. First, the study’s cross-sectional design limits the ability to determine causality when assessing the associations between EIH and the independent variables included in this study. Longitudinal and/or interventional research is needed to better understand whether changes in these or other factors may enhance the EIH response in people with knee OA. Second, the order of the pre-exercise tests, including the questionnaires, was not randomised, so we cannot rule out the influence of test order bias. Third, this exploratory secondary analysis was not designed with a priori power calculations for the specific associations examined. With 119 participants and 13 predictors in our models, we likely had adequate power to detect moderate-to-large effects but limited power for small effects that could still be clinically meaningful. Non-significant results should not be interpreted as evidence of no association, but rather as associations not reliably detected with our sample size. The modest proportion of variance explained (Figure 3) underscores the exploratory nature of these findings and the need for replication in adequately powered confirmatory studies with prespecified hypotheses. Fourth, as an analysis of baseline data from volunteers participating in a randomised controlled trial with specific inclusion and exclusion criteria, the findings may not be broadly generalisable to all people living with knee OA. In particular, no routine musculoskeletal imaging (X-ray or MRI) and scoring was obtained as part of this study, so we could not examine associations between structural disease severity (e.g., Kellgren–Lawrence grade, cartilage thickness, synovitis) and EIH. Additionally, all participants had established OA, with a median pain duration of 4 years. The absence of an early-stage OA cohort limits generalisability and supports the need for prospective studies in earlier disease stages. Finally, it is important to emphasise that the results of this study only relate to EIH induced by a sustained, submaximal isometric contraction. As such, it remains possible that EIH, and the factors contributing to its variability, may be different with other types of exercise. 

## 5. Conclusions

Older age, lower pre-exercise anxiety, and expecting less exercise-induced pain were associated with larger EIH following isometric exercise in people with knee OA. Although the explained variance was modest, identifying the limited influence of these psychological and neurophysiological factors provides an empirical basis for refining mechanistic models of EIH. The substantial proportion of unexplained variance underscores the complexity of pain modulation and highlights the need to investigate additional potential contributors, such as autonomic function, immune reactivity, molecular biomarkers, and physical activity level.

Clinically, the findings suggest that interventions targeting pre-exercise expectations and anxiety may enhance the pain-relieving effects of exercise, providing a rationale for embedding expectation-management and reassurance strategies within exercise therapy for knee OA.

## Figures and Tables

**Table 1:** Participant characteristics and measures (*n* = 119). Data are presented as mean (SD) or median (IQR) unless otherwise stated.

Age (y)	68	(10)
Sex (females (%))	53	(45%)
Height (cm)	170	(10)
Weight (kg)	82	(16)
BMI (kg/m^2^)	28	(25–31)
Ethnicity: frequency/percentage		
New Zealand European	101	(85%)
New Zealand Māori	7	(6%)
Tongan	1	(1%)
Chinese	2	(2%)
Indian	2	(2%)
Other	6	(5%)
Duration of knee pain (months)	48	(24–120)
LLTQ (0–100)	26	(6)
HADS-Depression (0–21)	4	(2–6)
HADS-Anxiety (0–21)	5	(3–7)
TSK-11 (11–44)	25	(5.1)
PCS (0–52)	10	(5–17)
BPI-Ave (0–10)	4	(3–5)
BPI-Worst (0–10)	8	(4–10)
BPI-Least (0–10)	3	(1.5–4)
BPI-Interference (0–10)	4	(2)
CSI (0–100)	45	(20–60)
mTS	30	(10)
CPM	−4	(−15–3)
Peak pain conditioning stimulus (0–100)	58	(45–70)
OffA	−19	(17)
Peak Torque (Nm)	117	(91–116)
EIH testing order (knee first (%))	57	(48%)
Expected change in knee pain (0–100)	45	(20–60)
Actual change in knee pain (0–100)	0	(0–2)
Maximum knee pain during contraction (0–100)	10	(0–50)
Time to failure (s)	300	(246–300)
Max RPE (6–20)	19	(17–20)

Abbreviations: BMI, Body Mass Index; BPI, Brief Pain Inventory; CSI, Central Sensitisation Index; CPM, Conditioned Pain Modulation; HADS, Hospital Anxiety and Depression Scale; IQR, Interquartile Range; LLTQ, Lower Limb Task Questionnaire; m, metres; min, minutes; OffA, offset analgesia; PCS, Pain Catastrophising Scale; s, seconds; SD, Standard Deviation; TSK, Tampa Scale for Kinesiophobia; TS, Temporal Summation.

**Table 2:** Pressure pain threshold (PPT), absolute EIH (EIH_abs_), and relative EIH (EIH_rel_) values at the knee and forearm with sustained submaximal isometric exercise. Values are displayed as median (interquartile range).

Test Site	PPT Pre-Exercise	PPT Post-Exercise	EIH_abs_ (kPa)	EIH_rel_
Knee	252 (176–353)	293 (190–413) *	28 (1–93)	1.12 (1.01–1.35)
Forearm	249 (188–356)	251 (199–388) *	12 (−19–58)	1.06 (0.91–1.22)

* Represents significant within group change from pre- to post-exercise *p* < 0.01.

## Data Availability

Data available following reasonable request.

## Abbreviations

The following abbreviations are used in this manuscript:

BMI	Body Mass Index
BPI	Brief Pain Inventory
BP	Blood Pressure
CCK	Cholecystokinin
CPM	Conditioned Pain Modulation
CSI	Central Sensitisation Inventory
DNIC	Diffuse Noxious Inhibitory Control
EIH	Exercise-Induced Hypoalgesia
EIHabs	Absolute Exercise-Induced Hypoalgesia
EIHrel	Relative Exercise-Induced Hypoalgesia
eVAS	Electronic Visual Analogue Scale
ExBelief	Exercise Belief (Pain can be reduced from just a single session of exercise)
HADS	Hospital Anxiety and Depression Scale
IL-6	Interleukin 6
IQR	Interquartile Range
ISI	Inter-Stimulus Interval
LLTQ	Lower Limb Task Questionnaire
mTS	Mechanical Temporal Summation
MVIC	Maximum Voluntary Isometric Contraction
NSAIDs	Non-Steroidal Anti-Inflammatory Drugs
NPRS	Numerical Pain Rating Scale
OA	Osteoarthritis
OffA	Offset Analgesia
PPT	Pressure Pain Threshold
PPTpre	Pre-Exercise Pressure Pain Threshold
PPTpost	Post-Exercise Pressure Pain Threshold
PCS	Pain Catastrophising Scale
RPE	Rating of Perceived Exertion
SD	Standard Deviation
TSK	Tampa Scale of Kinesiophobia
TS	Temporal Summation
